# In Vitro Characterization of Lactic Acid Bacteria from Indonesian Kefir Grains as Probiotics with Cholesterol-Lowering Effect

**DOI:** 10.4014/jmb.1910.10028

**Published:** 2020-01-23

**Authors:** Dandy Yusuf, Lilis Nuraida, Ratih Dewanti-Hariyadi, Dase Hunaefi

**Affiliations:** 1 Department of Food Science and Technology, Faculty of Agricultural Technology, IPB University (Bogor Agricultural University), Bogor, West Java, Indonesia; 2 Southeast Asian Food and Agricultural Science and Technology (SEAFAST) Center, IPB University (Bogor Agricultural University), Bogor, West Java, Indonesia

**Keywords:** Cholesterol, kefir grains, lactic acid bacteria, *Lactobacillus*, probiotics

## Abstract

Indonesian kefir grains are potential sources of lactic acid bacteria (LAB) that may act as probiotics with specific functional properties. In this study we explored the potential of the probiotic and cholesterol-lowering effect of LAB isolated from Indonesian kefir grains obtained from Bogor, Bandung, Jakarta, and Yogyakarta. The results revealed that 10 isolates showed considerable survivability at low pH and bile salt with total cell reduction of ~3 log colony-forming units per milliliter after exposure to pH 2.5 and 0.5% (w/v) bile salt for 1 and 3 h, respectively. All strains exhibited strong antimicrobial activities against pathogenic bacteria and were sensitive to a wide spectrum of antibiotics but exhibited weak bile salt hydrolase activity. Identification based on 16S RNA suggested that nine isolates were *Lactobacillus kefiri* and one was *Lactobacillus rhamnosus*. The ability of the isolates to reduce cholesterol from the media varied, ranging from 22.08% to 68.75% with the highest reduction shown by *L. kefiri* JK17. The ability to remove cholesterol from the media decreased greatly in resting and dead cells, ranging from 14.58% to 22.08% in resting cells and from 7.89% to 18.17% in dead cells. It can be concluded that Indonesian kefir grains contain LAB potentially acting as probiotics capable of reducing cholesterol. The cholesterol-lowering effect especially occurs when the cells are metabolically active.

## Introduction

Kefir grain is a starter culture for the production of kefir. The grain is densely populated by lactic acid bacteria (LAB) species, acetic acid bacteria, and yeast. Kefir is reported to have many health benefits, such as prevention of lactose intolerance [1], antimicrobial [2] and antioxidant [3] effects, reduction of cholesterol [4], and prevention of diabetes [5]. It is suspected that these benefits originate from the presence and activity of LAB, which can act as probiotics [6] and/or agents producing bioactive compounds [7].

The Food and Agriculture Organization (FAO)/World Health Organization (WHO) defines probiotics as “living microorganisms that if consumed in sufficient quantities are able to provide health benefits to their host.” Some of the requirements for probiotic bacteria are that they be generally recognized as safe (GRAS), have a history of use in food, and are able to survive in the upper gastrointestinal tract, namely, exposure to gastric acid pH (~2.5) and bile salt (~0.5%) for 1 to 3 h. In addition, probiotics should be sensitive to antimicrobials or antibiotics [8].

The WHO (2015) estimated that 17.5 million deaths occur annually because of cardiovascular disease. Cardiovascular disease is a non-communicable disease that can be caused by high cholesterol content in blood serum [9]. Many probiotic strains of LAB have been reported to possess cholesterol-reducing activity [10, 11]. *Lactobacillus kefiri* DH5 is one of the LAB from kefir, which has demonstrated probiotic properties and is able to reduce cholesterol [12]. The cholesterol-reducing activity in probiotics is strain dependent. The mechanisms of cholesterol reduction by probiotics include adsorption or attachment of cholesterol on the cell surface [13], assimilation or incorporation of cholesterol into the cellular membrane [14], incorporation and conversion to coprostanol [15], hydrolysis of glycine- or taurine-conjugated bonds to amino acids and free bile salts [16], and enzymatic de-conjugation by bile salt hydrolase (BSH) [17]. Studies on cholesterol removal by probiotic bacteria revealed that probiotic bacteria removed cholesterol through several different mechanisms [11, 14, 18, 19].

Traditionally, dairy products and their derivatives are the main source of LAB; however, currently, LAB have been isolated from traditional fermented food, meat, plants, humans, insects, soil, and marine creatures, and many of them showed probiotic characteristics [20, 21]. *Lactobacillus* and *Bifidobacterium* are the most widely used groups of probiotic bacteria in food [8]. In previous studies, we have succeeded in isolating 30 LAB from Indonesian kefir grains obtained from Bogor, Bandung, Jakarta, and Yogyakarta (report in the process of publication). It is suspected that among these LAB isolates there are LAB with probiotic properties of cholesterol-lowering effect. The objectives of this study were to evaluate the potential probiotic properties of LAB from Indonesian kefir grains, identify the isolates, and evaluate their cholesterol-lowering effect.

## Materials and Methods

### LAB Isolates

A total of 30 LAB isolates, including both rods and spheres, were obtained from four Indonesian kefir grains obtained from Bogor, Bandung, Jakarta, and Yogyakarta.

### Growth in Media Containing Bile Salt

This test was performed for the initial selection of isolates capable of surviving bile salt. The inoculum was prepared by growing LAB at 37°C for 24 h, and then as much as 1% was inoculated into de Man, Rogosa and Sharpe (MRS) broth supplemented with 0.5% (w/v) bile salt (Oxgall; USA) and incubated at 37°C for 6 h. Absorbance at 0 and 6 h was measured using an ultraviolet-visible (UV-Vis) spectrophotometer (A_600_; Shimadzu, Japan). An increase in absorbance indicates cell growth.

### Evaluation for Probiotic Properties

**Acid and bile salt tolerance.** Acid and bile salt tolerance was evaluated on the basis of the strains’ survival after incubation at pH 2.5 and bile acid [10]. The inoculum was prepared by centrifugation (10,000 ×*g*, 5 min, 4°C) of 1 ml LAB culture in MRSB for 24 h. For acid tolerance assay, cell pellets were re-suspended in phosphate-buffered saline (PBS; Oxoid) acidified using 0.1 N HCl to pH 2.5. As for the bile salt tolerance assay, cell pellets were re-suspended in PBS supplemented with 0.3% (w/v) bile salt. Incubation was done at 37°C for 1 h for acid tolerance and 3 h for bile salt tolerance assays. Enumeration of LAB counts at 0 h and after incubation was carried out on MRS agar at 37°C for 48 h. The difference in the number of colonies before and after incubation shows a decrease in the number of LAB due to acid or bile salt.

**Antimicrobial activity.** The antimicrobial activity of the LAB isolates was tested using the well diffusion method [22] against *Salmonella typhimurium*, *Escherichia coli*, and *Bacillus cereus* in Mueller–Hinton agar (Difco, USA).

**Antibiotic resistance.** Antibiotic resistance was tested for ampicillin (AMP), chloramphenicol (CHL), tetracycline (TET), and streptomycin (STR) [23], under compliance with the European Food Safety Authority (EFSA) [24] technical guidelines.

### BSH Activity

Qualitative BSH activity evaluation was done by observing a precipitation zone after incubation of spotted culture on MRSA supplemented with 0.5% (w/v) sodium salt of taurodeoxycholic acid and 0.37 g/l CaCl_2_ [10].

### Identification of LAB Isolates Based on 16S rRNA Gene Sequence

**DNA extraction.** Cell suspension was prepared by growing the isolate in MRSB supplemented with 0.1%glycine and incubated at 37°C for 24 h. DNA extraction was done according to the manufacturer's instruction using a DNA extraction kit (Promega, USA).

**Amplification.** Amplification of the 16S rRNA gene was carried out by polymerase chain reaction (PCR) using forward primer 27F (5’-AGAGTTTGATCCTGGCTCAG-3’) and reverse primer 1492R (5’-GGTTACCTTGTT ACGACTT-3’) [25]. The PCR mixture consisted of 15 μl GoTaq Green Master Mix (Promega), 0.6 μl primers (10 μmol), 2 μl template, and 11.8 μl nuclease-free water. The final volume of the PCR mixture was 30 μl. The PCR (Takara Thermal Cycler Dice Gradient, Japan) condition was as follows: pre-denaturation at 95°C for 5 min, denaturation at 95°C for 1 min in 35 cycles, attachment at 58°C for 2 min, elongation at 72°C for 2 min, and final extension at 72°C for 10 min.

**Identification.** The PCR products were then run on agarose gel electrophoresis. As much as 4 μl PCR products were mixed with loading dye (1 μl) and then placed in 1% (w/v) agarose well in Tris-acetate-EDTA buffer solution. Electrophoresis was carried out at a voltage of 90 V for 45 min, stained with ethidium bromide dye, and visualized using Gel Documentation System (Atto Corp., Japan) under UV light. The PCR products were sent to 1^st^ Base Laboratories (Malaysia) for sequencing. The sequencing data were then analyzed on MEGA7 software version 7.0, and the homology was searched through the Basic Local Alignment Search Tool (BLAST) on the National Center for Biotechnology Information website. A phylogenetic tree based on 16S rDNA genes was then constructed.

### Cholesterol-Lowering Effect of Growing, Resting, and Dead Cells

For evaluation of growing cells [10], LAB were grown in MRSB containing 0.5% bile salt at 37°C for 24 h. The cell pellet was separated by centrifugation (10,000 ×*g*, 5 min, 4°C) and inoculated into MRSB + 0.5% bile salt + 100 ppm cholesterol (PEG-600; Sigma, USA). The mixture was then incubated at 37°C for 24 h. For evaluation of resting cells, the cell pellet was inoculated into PBS solution (pH 6.8) + 0.5% bile salt + 100 ppm cholesterol and then incubated at 37°C for 24 h. For evaluation of dead cells, the cell suspension in PBS was heated at 121°C for 15 min. The cell pellet was then separated by centrifugation and inoculated into MRSB + 0.5% bile salt + 100 ppm cholesterol. The mixture was then incubated at 37°C for 24 h. The effect of cell concentration (10× and 100× diluted cell suspension) of resting and growing cells on cholesterol removal was evaluated on the isolate with the highest removal ability.

### Analysis of Cholesterol Removal

The amount of cholesterol remaining in the medium was determined by O-phthalaldehyde (OPA) method [26] after cell separation by centrifugation (10,000 ×*g*, 5 min, 4°C) to obtain the supernatant. The determination of cholesterol from the cell-free supernatant was based on colorimetric method using OPA (Sigma) measured at 550 nm. Cholesterol concentration was determined using a standard curve. The percentage of cholesterol assimilation was determined by this formula: [Initial cholesterol concentration in the medium (ppm) − Concentration of cholesterol remaining in culture (ppm)] / Initial cholesterol concentration in the medium × 100%.

### Scanning Electron Microscopy (SEM) Analysis

SEM analyses were done on two representative isolates, *i.e.*, *L. kefiri* JK17 and *L. kefiri* YK4. The growing, resting, and dead cells were prepared according to the method in Section F. As a control, the isolates were inoculated into MRSB without cholesterol. After incubation was completed, the culture was centrifuged (10,000 ×*g*, 5 min, 4°C) and then rinsed twice with PBS solution (pH 7.0; Oxoid). The cholesterol attached to the cell wall was then observed using low-vacuum SEM (JSM-5310LV; Jeol Ltd., Japan).

## Results and Discussion

### Growth in Media Containing Bile Salt

The results showed that all 30 LAB isolates can grow in MRSB containing 0.5% bile salt. The increase in the absorption values varies with an average increase in absorbance of 63.66%. A total of 19 isolates whose increased absorbance values were above average were selected for further evaluation.

### Acid Tolerance

The 19 LAB isolates showed varying tolerance to pH 2.5, which is in agreement with various studies showing that the ability of LAB to survive low acid is strain dependent [27-29]. The number of cell decrease ranged between 0.57 and 2.04 log colony-forming units (CFU)/ml. The pH value of 2.5 correlated with human stomach acidity. Isolates BG8, BG9, BG13, BD4, JK1, JK6, JK17, JK19, YK4, and YK7 showed a considerable tolerance to low pH as shown by slight decreases in viable cells (Table 1). Isolate JK1 was the most acid tolerant, with a decrease of 0.57 log CFU/ml. Low acidity damages the cytoplasmic membrane and destroys the intracellular components of the cell, but some strains have stronger cytoplasmic membrane permeability and ability to adapt to low acidity [30]. Ten isolates with slight decrease in viable cells were then selected for further evaluation.

### Bile Salt Tolerance

The results suggested that the 10 LAB isolates that were most resistant to low pH showed various tolerance to 0.3% bile salt. The number of cell decrease ranged between 0.69 and 1.59 log CFU/ml (Table 1). JK17 was the most tolerant to bile acid with a cell decrease of 0.69 log CFU/ml. The ability to tolerate bile salt is assumed to be associated with proteins or enzymes produced by LAB, which influence cell membrane, cell homeostatic properties, or change in the structure of bile salt [31]. Several LAB strains have been reported to produce BSH enzyme that can hydrolyze conjugated bile salt into deconjugated bile salt and decrease the toxicity level to the cells [32]; however, the present isolates were not strong BSH producers.

JK17 was the most prospective isolate, with a total decrease of 1.69 log CFU/ml after exposure to bile salt and low pH (2.5; Table 1). The number of probiotics that are able to survive low pH and bile acid will determine the effectiveness of the probiotic. The dosage of 10^6^ to 10^9^ CFU/ml viable organisms reaching the intestinal tract in humans is considered as the most effective [33]. When it is assumed that the probiotic organism in the product is 109 CFU/ml, the decrease up to 103 CFU/ml would be tolerable. Therefore, the 10 isolates obtained in the present research were considered as potential probiotics as shown by the total decrease of viable cells after exposure to low pH and bile acid (Table 1).

### Antimicrobial Activity

**Fig. 1:**
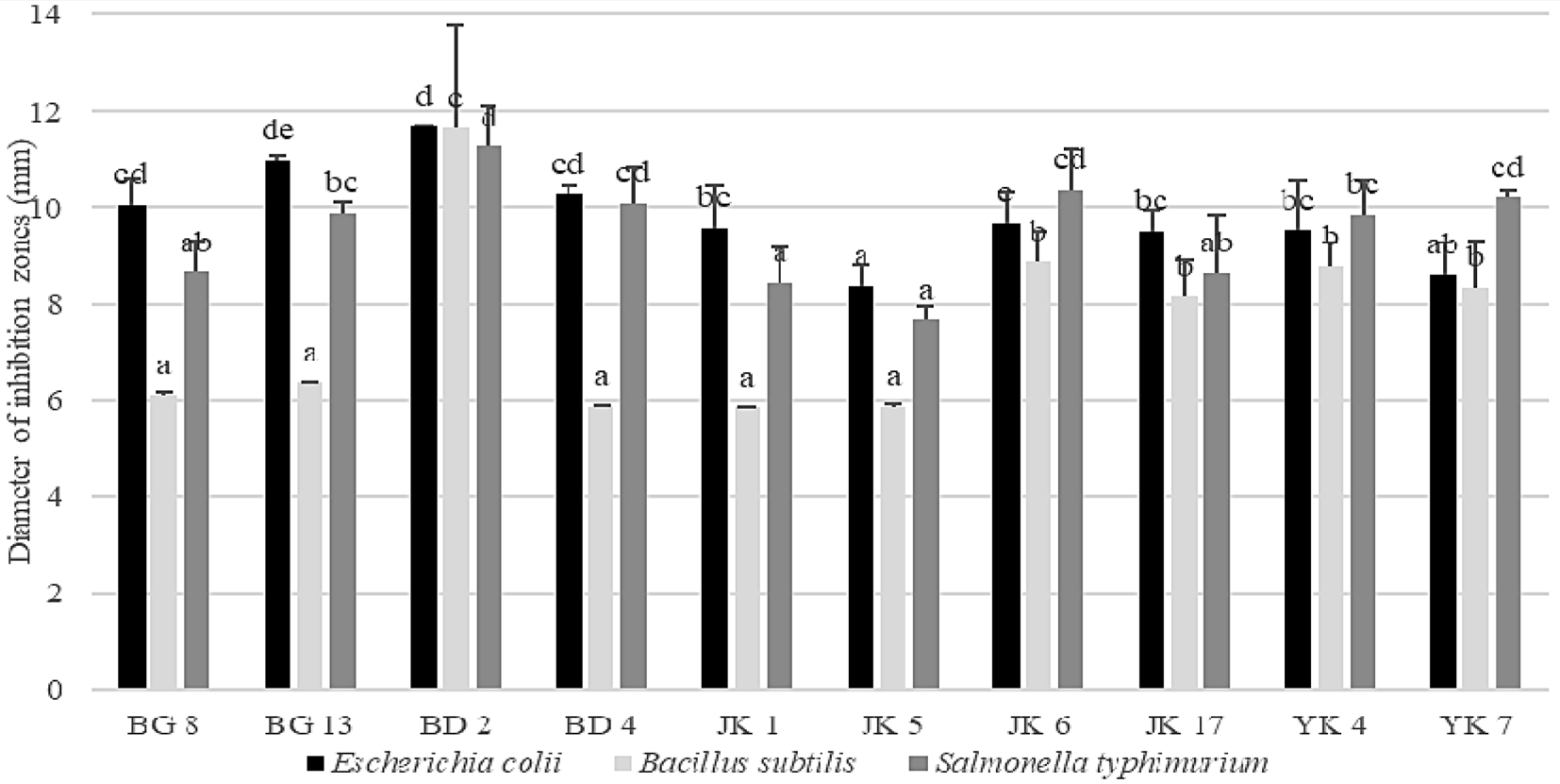
Antimicrobial activity of LAB isolated from Indonesian kefir grains. Different superscript letters are significantly different (*p* < 0.05) by Duncan’s multiple range test.

The results of the experiment indicate that the 10 LAB isolated from Indonesian kefir grains showed various antimicrobial activities (Fig. 1). *Lactobacillus rhamnosus* BD2 showed the greatest antimicrobial activity. Antimicrobial substances produced by LAB have been widely reported, including lactic acid, bacteriocin, reuterine, and reutericycline [21]. Lactic acid is the main product of LAB, so it is thought to be the major inhibitor. Although the isolates capable of producing bacteriocin, reuterine, or reutericycline are still unknown, it is necessary to carry out further research on the identification of antimicrobial substances produced by the isolates.

### Antibiotic Resistance

The 10 isolates were sensitive to AMP, CHL, TET, and STR. The minimum inhibitory concentrations of antibiotics that inhibited the isolates were below the limit concentration set by EFSA for *L. rhamnosus* and *L. kefiri*. Antibiotic resistance is one of the safety indicators recommended by the FAO/WHO to be tested on probiotic candidates. This is due to the potential of probiotics to transfer antibiotic-resistant genes to other bacteria in the digestive system [34]. The antibiotics tested in this study are wide-spectrum commercial antibiotics, which inhibit gram-positive and gram-negative microbes [24].

### BSH Activity

The 10 isolates tested in this study showed weak BSH activity as indicated by the thin precipitation zone compared with controls. The benefit of BSH production by probiotic bacteria is still under discussion. BSH produced by probiotics can help reduce host cholesterol [35], but some other researchers suggested that the activity of BSH probiotics is not entirely beneficial, because it is suspected that conjugated bile salts can be toxic compounds for the host, which can interfere with intestinal microbiota and cause digestive disorders [31].

### Identification of 16S rRNA Gene Sequencing

**Fig. 2:**
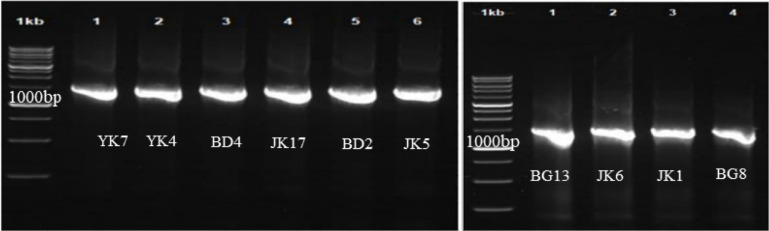
PCR results of 16S rRNA gene on agarose gel of 10 LAB isolated from kefir grains.

The PCR results of 16S rRNA of the isolates on agarose gel electrophoresis are shown in Fig. 2. The amplicon size was 1,450 bp. The results of BLAST suggested that nine isolates had similarities of more than 99% with *L. kefiri* and one isolate had 99.79% similarity with *L. rhamnosus*. A similarity of more than 94% suggested high homology, and the samples were of the same species [36].

**Fig. 3:**
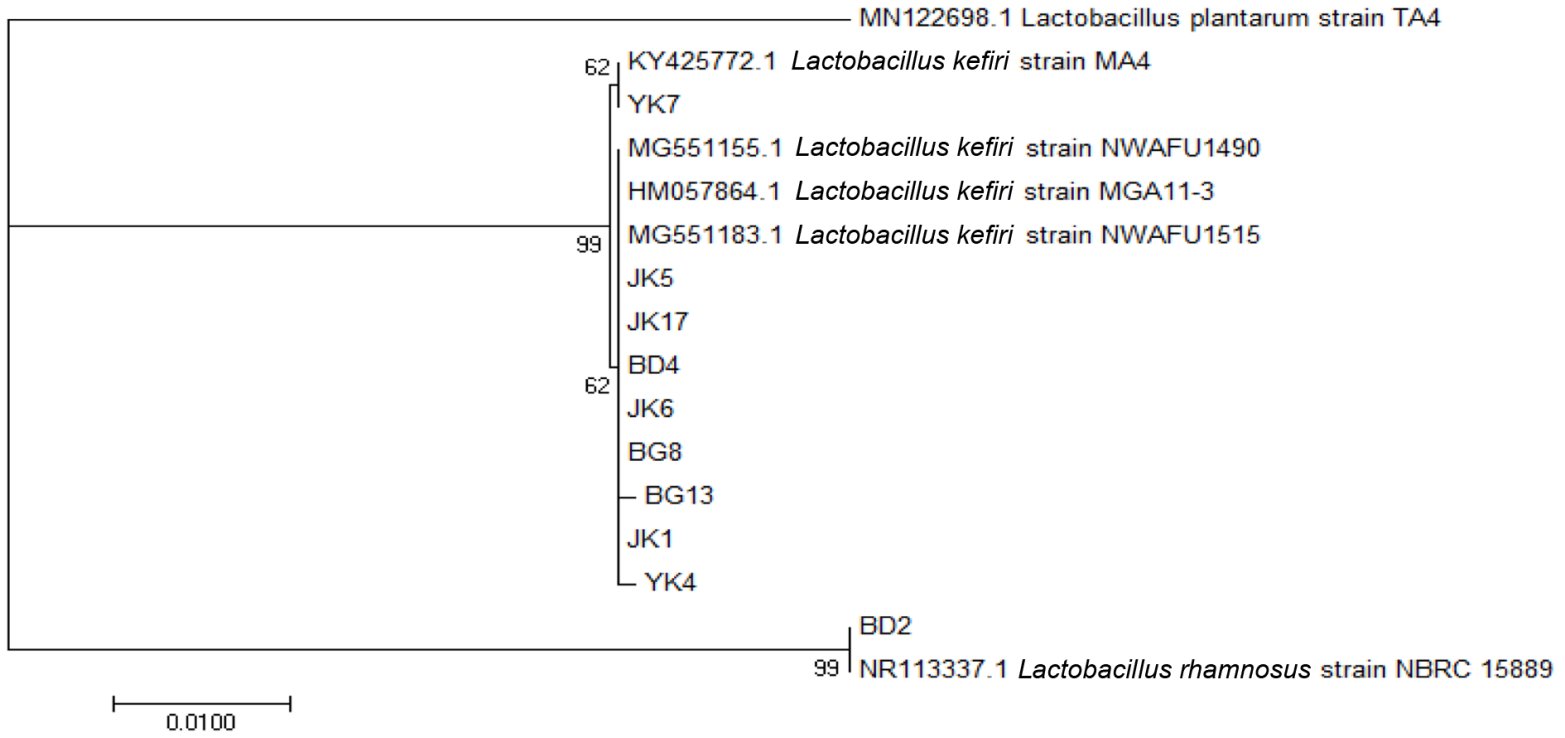
Phylogenetic tree based on the homology of 16S rRNA gene sequence of 10 LAB isolated from Indonesian kefir grains.

The phylogenetic tree of the 10 isolates formed two branches, namely, *L. kefiri* and *L. rhamnosus* with *Lactobacillus plantarum* as the outgroup (Fig. 3). The bootstrap value in two branches showed the percentage of the accuracy of the branching, which was statistically calculated to be as many as 1000 repetitions [37]. The bootstrap value showed that the divergences or separation by these branches were correct or reliable [38].

### Cholesterol Removal by Growing, Resting, and Dead Cells

Growing, resting, and dead cells of the 10 LAB isolates were able to remove cholesterol from the medium (Table 2). Compared with resting and dead cells, growing cells reduced cholesterol the most. The greatest cholesterol reduction was observed in *L. kefiri* JK17 (*i.e.*, ~68.75%). The value showed a significant difference (*p* < 0.05) compared with other strains, including *L. rhamnosus* R23 positive control [39]. Although the ability in removing cholesterol from the media decreased greatly in resting and dead cells, the reduction of cholesterol by resting cells was still higher than that by dead cells.

The results of this study confirm that cholesterol removal activity is strain dependent as shown by various degrees of reduction among *L. kefiri*. LAB cells in the growth phase have the greatest cholesterol removal effect. Similar results were also reported by other researchers on *Lactobacillus* isolated from fermented food [14], LAB from fermented mustard [18], and *Lactococcus lactis* subsp. *lactis* [19]. Growing cells were suggested to have the ability to assimilate and absorb cholesterol as fatty acid for constructing cell membranes [40] and converting cholesterol to coprostanol [15]. Cholesterol uptake by growing cells causes the difference in levels of cholesterol removal from the medium [18]. For in vitro study, the most suggested mechanism of cholesterol removal is assimilation or incorporation of cholesterol into cells and adhesion of cholesterol on the cell surface [14]; however, the present study showed only a small degree of cholesterol removal by resting and dead cells, suggesting that the main mechanism was uptake by growing cells. The present results suggested that high cholesterol removal is growth associated and might depend on the metabolic process. When growth ceases, the ability to remove cholesterol significantly decreases. Our results were different from previous report [10] that growing, resting, and dead cells of *L. plantarum* EM showed high cholesterol removal.

The present results also showed that when resting cells were diluted by 10× (about 8 log CFU/ml), the cholesterol removed was significantly lower as compared to undiluted cells (10^9^ CFU/ml; Table 3). However, cholesterol removed by 100× dilution of resting cells was not significantly different from that by 10× dilution. There was no significant difference on the effect of biomass concentration on the cholesterol removed by the dead cells. The results support that the mechanism of cholesterol removal from the media by the isolates may involve metabolism and not depend on cell surface attachment.

### SEM Analysis

**Fig. 4:**
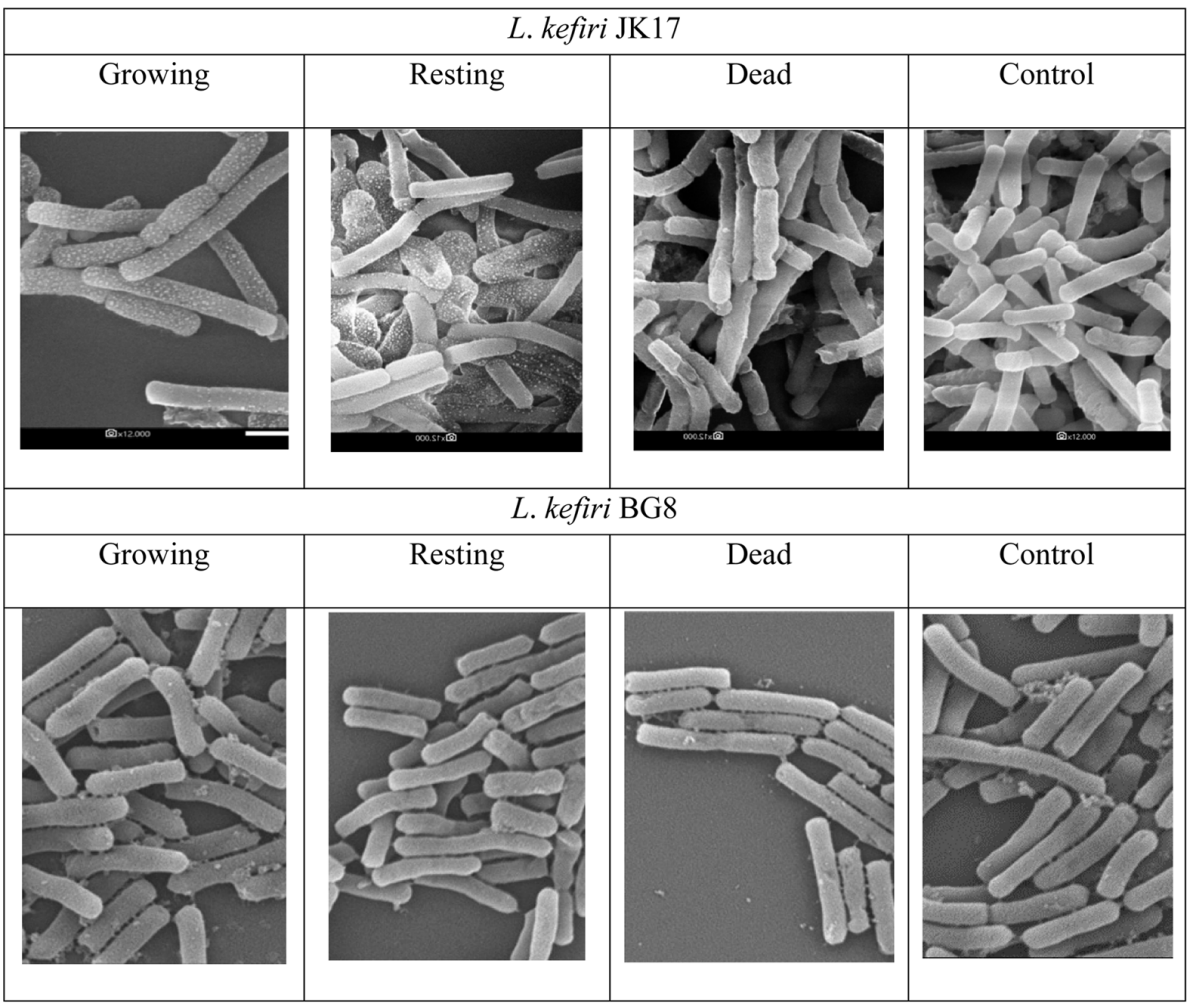
SEM images of *L. kefiri* JK17 and *L. kefiri* BG8 after incubation in media containing cholesterol (magnification of 12,000×).

SEM observation showed that cholesterol also attached to the cell surface (Fig. 4). The most attachment of cholesterol was shown on growing cells of *L. kefiri* JK17 compared with resting and dead cells. A small degree of cholesterol attached to the resting cells of *L. kefiri* JK17; however, almost no cholesterol was attached to the cell surface of dead cells, many of which appeared to be damaged. *L. kefiri* BG8 reduced cholesterol the least (Table 2). SEM observation showed that only a limited amount of cholesterol attached on the cell surface of *L. kefiri* BG8 (Fig. 4) and almost no cholesterol attached to resting and dead cells of *L. kefiri* BG8. SEM evaluation supported that cholesterol removal by LAB isolated from Indonesian kefir grain depends on the metabolic process of the live cells. Attachment on the cell surface was not the main the mechanism of cholesterol removal in the present study. Removal of cholesterol through several mechanisms has also been reported by other researchers [11, 14, 18, 19].

The present study revealed that nine *L. kefiri* strains and one *L. rhamnosus* strain isolated from Indonesian kefir grains meet the prerequisite criteria to be candidate probiotic bacteria, such as acid and bile tolerance, antimicrobial activity, and sensitivity to antibiotics. The 10 strains also showed cholesterol-lowering effect in growing or metabolic active state but much lower in resting and dead cells. *L. kefiri* JK17 is considered as the probiotic candidate having the best potential with the the specific function of cholesterol-lowering effect, especially in growing state. Removal of cholesterol by the isolate is expected to reduce cholesterol availability for absorption from the intestine into the blood. Therefore, further in vivo studies are required to confirm the isolate’s probiotic and cholesterol-lowering effect. The elucidation of the mechanism of how *L. kefiri* JK17 removes cholesterol is also an area that needs to be pursued.

## Figures and Tables

**Table 1:** Acid and bile salt tolerance of LAB isolated from Indonesian kefir grains.

Strains	Decrease of viable cells after 1 h at pH 2.5	Decrease of viable cells after 3 h at 0.3% bile salt	Total decrease of viable cells

	(log CFU/ml)		
BG8	1.53^ab^	1.12^ab^	2.65
BG13	1.69^ab^	1.59^abc^	3.28
BD2	2.04^a^	1.07^a^	3.11
BD4	1.27^b^	1.29^abc^	2.56
JK1	0.57^ab^	1.40^ab^	1.97
JK5	1.91^ab^	1.16^bc^	3.07
JK6	1.28^ab^	1.50^bc^	2.78
JK17	1.00^b^	0.69^bc^	1.69
YK4	1.30^b^	0.96^bc^	2.26
YK7	1.09^b^	0.99^c^	2.08

Means in the same column with different superscript letters are significantly different (*p* < 0.05) by Duncan's multiple range test.

**Table 2:** Cholesterol removal from the medium by LAB isolated from Indonesian kefir grains.

LAB isolates	Cholesterol reduced from the medium after 24 h (%)		

	Growing cells	Resting cells	Dead cells
*L. kefiri* BG8	22.08^eEF^	16.67^aEF^	15.11^aEF^
*L. kefiri* BG13	28.33^deDE^	17.92^aF^	12.33^aEF^
*L. rhamnosus* BD2	53.75^bB^	20.42^aEF^	16.69^aEF^
*L. kefiri* BD4	36.25^cdCD^	16.25^aF^	10.33^aEF^
*L. kefiri* JK1	42.50^bcBC^	14.58^aF^	7.89^aEF^
*L. kefiri* JK5	42.50^bcBC^	16.67^aEF^	12.75^aEF^
*L. kefiri* JK6	37.50^cdCD^	18.33^aEF^	14.83^aEF^
*L. kefiri* JK17	68.75^aA^	22.08^aEF^	15.81^aEF^
*L. kefiri* YK4	46.67^bcBC^	21.67^aEF^	18.17^aEF^
*L. kefiri* YK7	36.25^cdCD^	17.92^aEF^	12.06^aEF^
*L. rhamnosus* R23	20.83^eEF^	14.17^aF^	10.25^aEF^

Means with different superscript letters in the same column (a–d) and superscript capital letters in the same row (A–F) are significantly different (*p* < 0.05) by Duncan’s multiple range test.

**Table 3:** Cholesterol removal by different cell concentrations of resting and dead cells of *L. kefiri* JK17.

Cell conditions	Cholesterol reduced from the medium (%)		

	9 CFU/ml^*^	8 CFU/ml	7 CFU/ml
Resting cells	22.08^a^	13.75^b^	10.00^bc^
Dead cells	15.81^a^	15.00^ab^	11.25^b^

^*^The viable cell concentration before incubation as resting cells and before heat killing was 1.26 × 10^9^ CFU/ml. Means in the same row with different superscript letters are significantly different (*p* < 0.05) by Duncan’s multiple range test.
